# Magnetic Resonance Cholangiography Diagnosing Post-cholecystectomy Biliary Injuries

**DOI:** 10.7759/cureus.56475

**Published:** 2024-03-19

**Authors:** Maria Katherinne Florez Leguia, Brayan Muñoz-Caicedo, Johan Sebastian Lopera Valle, Brian Daniel Noreña Rengifo, Astrid Arroyave Toro, Vanessa García Gómez

**Affiliations:** 1 Department of Radiology, Division of Body Imaging, Clínica CES, Medellín, COL; 2 Department of Radiology, Universidad de Antioquia, Medellín, COL; 3 Department of Interventional Radiology, San Vicente Fundación, Medellín, COL; 4 Department of Radiology, Division of Body Imaging, San Vicente Fundación, Medellín, COL; 5 Department of Radiology, Division of Body Imaging, Hospital Pablo Tobón Uribe, Medellín, COL

**Keywords:** post-cholecystectomy, cholecystectomy, biliary fistula, magnetic resonance cholangiography, intraoperative complication

## Abstract

Objective: This study aimed to determine the diagnostic performance of contrasted magnetic resonance cholangiography for detecting bile duct lesions following cholecystectomy.

Materials and methods: A retrospective case series study was conducted that included patients over 18 years of age with suspected bile duct injury after cholecystectomy, who underwent contrasted magnetic resonance cholangiography, and who also had endoscopic retrograde cholangiopancreatography, surgery, or subsequent clinical follow-up. The images were interpreted by two radiologists who assigned the type of lesion according to the Strasberg classification. Qualitative variables were represented by frequencies and proportions, while quantitative variables were described using measures of central tendency and dispersion. Sensitivity, specificity, and predictive values were assessed, along with interobserver variability, using the kappa index.

Results: We included 20 patients with a median age of 51.5 years (interquartile range: 35), and 14 (70%) were women. In all 20 patients, lesions were identified on magnetic resonance cholangiography, of which 19 were confirmed with the gold standard for a positive predictive value of 100% (hepatobiliary-specific contrast agents) and 92% (extracellular contrast). The most frequent lesions were Strasberg E2 and E4 in five patients each. The kappa index was 1 in determining the presence or absence of bile duct injury and 0.9 in the Strasberg classification.

Conclusion: Contrasted magnetic resonance cholangiography is a method with high positive predictive value and almost perfect interobserver agreement for diagnosing bile duct lesions after cholecystectomy.

## Introduction

Cholecystectomy ranks among one of the most common surgical procedures, with approximately 60,000 cases carried out annually in Colombia alone, and it is the most common laparoscopic procedure [1]. Despite its frequency, bile duct lesions represent a significant and concerning complication following both open and laparoscopic cholecystectomy, with an estimated incidence ranging from 0.1% to 0.92%, being more frequent in the latter group [2-6]. These lesions entail considerable morbidity, with rates escalating up to 8.8%, and require complex management strategies [2-4]. Consequently, patients requiring surgery to address bile duct lesions as a complication of cholecystectomy incur a substantial 126% increase in healthcare service costs and a decrease in quality of life [3,7].

Endoscopic retrograde cholangiopancreatography (ERCP) is the gold standard for diagnosing bile duct lesions. However, it is an invasive procedure that can be associated with complications that increase morbidity and mortality [3]. For this reason, non-invasive imaging modalities such as magnetic resonance cholangiography (MR cholangiography or MRC), providing detailed anatomical and functional information, emerge as a crucial tool in evaluating the biliary tree in these patients [3,4,8-11]. T2-sequenced MRC is comparable to ERCP for evaluating bile duct anatomy. However, differentiating collections of biliary origin from other perihepatic collections is challenging. Traditional extracellular contrast mediums, with predominantly renal excretion, offer little specific information in the characterization of intra- and perihepatic lesions, unlike the hepatobiliary-specific contrast agents that allow the evaluation of the biliary tree anatomy and hepatic excretory function. This enables the precise identification of extravasations of the intrahepatic biliary system and the detection of perihepatic biliomas [12-16].

Therefore, the present study aimed to determine the diagnostic yield of contrasted MR cholangiography for detecting bile duct lesions after cholecystectomy over 24 months from January 2020 to December 2021 in two Colombian hospitals. It is a pioneering study in the local environment that aims to contribute evidence to this scarce and emerging topic which warrants further investigation in hospitals around the globe.

## Materials and methods

Study population

We performed a retrospective case series study involving patients over 18 years old who had suspected bile duct injury post-cholecystectomy and underwent contrasted MR cholangiography at Hospital Universitario San Vicente Fundación and Clínica León XIII from January 2020 to December 2021. Confirmation was done via ERCP, surgery, or follow-up for over 24 months. Patients with bile duct injuries due to trauma, lacking confirmation (ERCP, surgery, or follow-up), and MR cholangiography sequences of inadequate technical quality for precise interpretation were excluded.

MRI protocol

Contrasted MR cholangiographies were performed with the following parameters: Clínica León XIII (Siemens 1.5T resonator (Erlangen, Germany), contrast medium: Omniscan) and Hospital Universitario San Vicente Fundación (Ingenia Philips 1.5T and 3T resonators (Amsterdam, Netherlands), contrast medium: Dotarem, Prohance, or Primovist). A phased-array body coil was used. The patient was in a supine position, and the institutional protocols were followed, including the non-enhanced fat-suppressed 3D gradient recalled echo (GRE) axial T1-weighted imaging and the intravenous administration of 0.1 mmol/kg of contrast dose followed by a 20-mL saline infusion at a rate of 2 mL/s through a peripheral venous catheter with a minimum size of 20G. After that, contrasted 3D GRE axial T1-weighted imaging with fat suppression phases, arterial (30-45 sec post-contrast), venous (60-80 sec), late (80-180 sec), and hepatobiliary (30 min), were acquired. The late phase was also acquired in the coronal plane.

Parameters measured

Bile duct injury was defined as the presence of leaks, stenosis, complete transection, or excision of a segment with or without obstruction of the biliary tree, and its characterization was carried out through the Strasberg classification (Table 1) [8]. In cases where it was not possible to describe the injury using this classification, the term "unclassifiable" was used. The gold standard for diagnosis was ERCP, surgery, or subsequent clinical follow-up for patients not undergoing intervention.

**Table 1:** Strasberg classification of bile duct lesions.

Type	Description
Strasberg A	Bile drip from the cystic duct or gallbladder bed
Strasberg B	Section and occlusion of the biliary tree, commonly a right aberrant hepatic duct
Strasberg C	Dripping transection of a right aberrant hepatic duct
Strasberg D	Lateral injury of the main bile duct
Strasberg E	E1: Complete section of the bile duct more than 2 cm from the confluence
	E2: Complete section of the bile duct less than 2 cm from the confluence
	E3: Complete section of the bile duct at the level of the confluence
	E4: Complete section of the bile duct, leaving the hepatic ducts separated
	E5: Complete section of the bile duct or stenosis, including an aberrant duct

A search was conducted for contrasted MR cholangiography in each institution's Picture Archiving and Communication System (PACS). The MRC was performed in patients with clinical suspicion of bile duct injury after cholecystectomy over 24 months. Two radiologists, each specializing in body imaging with over 10 years of experience, independently interpreted these studies and classified the type of lesion using the Strasberg classification. In cases of disagreement, a third radiologist with 15 years of specialized experience in body imaging provided a blinded assessment as a tie-breaker. Radiologists were blinded to clinical and paraclinical information.

Statistical analysis

According to the proposed objectives, medical records were reviewed to determine the gold standard for subsequent analysis, which was carried out through the IBM SPSS Statistics for Windows, Version 22.0 (Released 2013; IBM Corp., Armonk, New York, United States). For descriptive purposes, absolute and relative frequencies were used to describe qualitative variables, while mean and standard deviation or median and interquartile ranges were used for the quantitative variables according to their distribution in the study population. A contingency or 2x2 table was constructed to compare the diagnostic test in question with the gold standard and thus obtain sensitivity, specificity, and predictive values. Interobserver variability was also calculated using the kappa index.

Ethics statement

The research obtained approval from the Ethics Committee of Hospital Universitario San Vicente Fundación and the Clínica León XIII (IN09-2020). This study was classified as a risk-free investigation, based on documentary research techniques, retrospective in nature, without interventions or modification of biological variables in the study population. In addition, it was conducted in adherence to ethical principles for research, following the Declaration of Helsinki and Resolution 008430 of 1993 from the Ministry of Health of Colombia.

## Results

**Figure 1:**
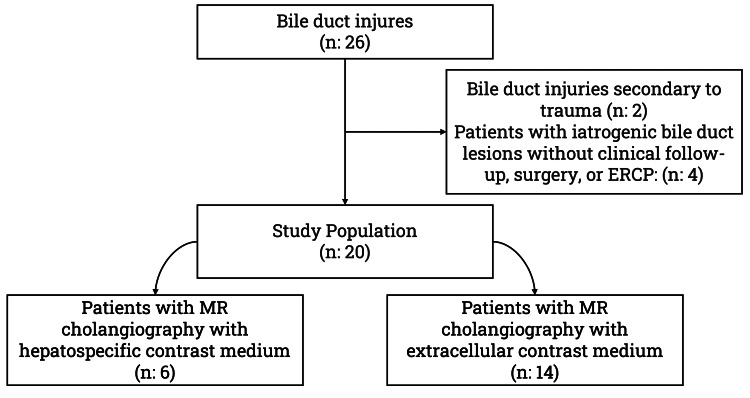
Study flowchart. ERCP: endoscopic retrograde cholangiopancreatography; MR: magnetic resonance

The search of patients diagnosed with bile duct injury over 24 months in the two high-complexity hospital institutions obtained a total of 26 patients, of which two corresponded to injury in the context of abdominal trauma and the remaining 24 to iatrogenic lesions after cholecystectomy. Of these 24, four patients who had not undergone ERCP or surgical intervention and for whom clinical follow-up was unavailable after discharge were excluded (Figure 1).

Thus, 20 patients with a median age of 51.5 years (interquartile range: 35) were included, of which 14 (70%) were women, and all were taken for cholecystectomy with a diagnosis of cholelithiasis. Fourteen cholecystectomies (70%) were performed laparoscopically, and the remaining six (30%) were performed by right subcostal laparotomy. Cholelithiasis with acute cholecystitis was present in 11 (55%) of the 20 patients, and in six (30%), there was an associated complication such as Mirizzi syndrome (three of these patients) (Table 2).

**Table 2:** Preoperative diagnosis and complications (n=20).

Population	n (%)
Men	6 (30)
Women	14 (70)
Pre-surgical diagnosis	n (%)
Cholelithiasis	20 (100)
Cholecystitis	14 (70)
Acute	11 (55)
Chronic	3 (15)
Associated findings	6 (30)
Mirizzi syndrome	3 (15)
Choledocholithiasis/pancreatitis	2 (10)
Gangrenous/perforated cholecystitis	2 (10)
Pyocolecyst	1 (5)

Bile duct injury was identified during cholecystectomy in three patients, while it was suspected in the remaining 17 (85%) due to postoperative symptoms of abdominal pain, bilioma, and jaundice in 10, five, and five patients, respectively.

Regarding MR cholangiography studies, 14 (70%) were performed with extracellular contrast medium (gadolinium) and six (30%) with intracellular contrast (gadoxetic acid). The gold standard was surgical hepatobiliary intervention in seven patients (35%), ERCP in seven patients (35%), and a combination of surgery plus ERCP in the remaining six patients (30%). 

**Figure 2:**
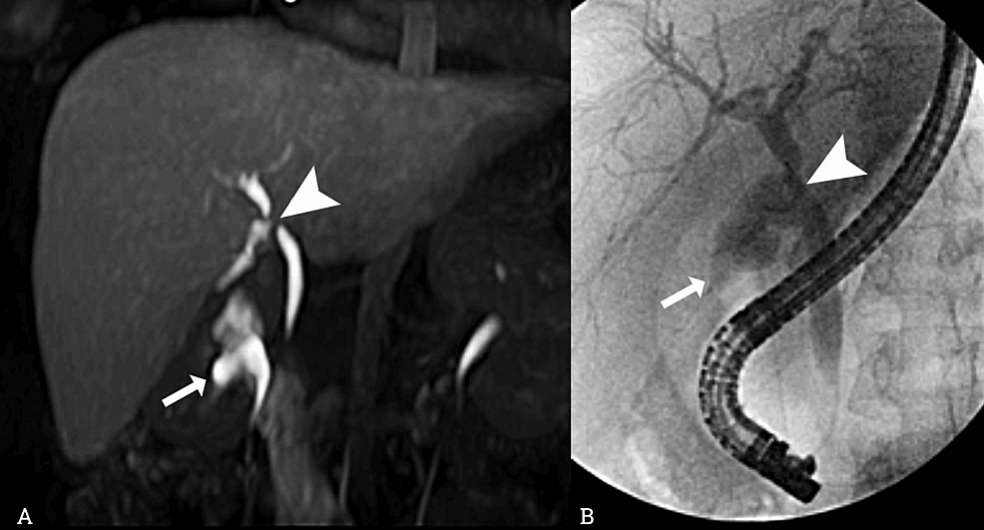
MR cholangiography and ERCP correlation findings. (A) MR cholangiography with hepatospecific contrast (gadoxetic acid), coronal acquisition in the hepatobiliary phase at 40 minutes, and high-intensity projection reconstruction. A biliary injury is 2 cm from the hepatic duct confluence (arrowhead) and contrast filtration to the subhepatic space (arrow). (B) ERCP image of the same patient confirming findings with contrast medium leaked from the biliary tree 2 cm from the confluence (arrowhead) due to bile duct injury Strasberg E2. ERCP: endoscopic retrograde cholangiopancreatography; MR: magnetic resonance

In all 20 patients, the presence of bile duct lesion was determined on MR cholangiography, of which 19 (95%) were confirmed with the gold standard and a false positive (study with extracellular contrast medium) was documented, for a positive predictive value (PPV) of 100% in studies with hepatospecific contrast and 92% for those with extracellular contrast (Figure 2). No MRI studies without bile duct injury were found. So, it was not possible to calculate sensitivity, specificity, and negative predictive values.

**Figure 3:**
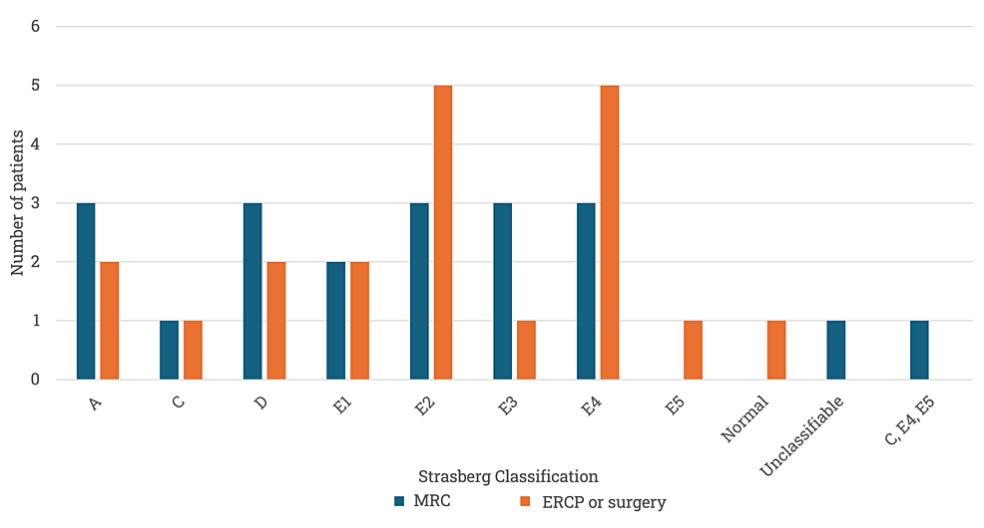
Frequency of Strasberg lesion types according to MR cholangiography and the gold standard (n=20). MRC: magnetic resonance cholangiography; ERCP: endoscopic retrograde cholangiopancreatography

Figure 3 shows the frequency of the types of lesions according to Strasberg, with the most frequent, according to the gold standard, being types E2 and E4 in five (25%) patients each. On the other hand, there was concordance of the type of lesion in 14 (70%) of the 20 patients when comparing the classification by MR cholangiography and ERCP/surgery. Of the six (30%) discrepancies, three were present in the studies performed with hepatospecific and three in the studies with extracellular contrast medium.

In one patient, it was not possible to classify the lesion by MRC or ERCP, being an E4 type according to surgery. In another patient who had all three procedures (MR cholangiography, ERCP, and surgery), ERCP presented a false negative in the characterization of an E4-type lesion adequately diagnosed in both MR cholangiography and surgery. The kappa index was 1 in determining the presence or absence of bile duct injury and 0.9 in the Strasberg classification. However, it should be noted that in the patient in whom there was discordance, the third radiologist who evaluated the images granted a third classification without achieving the tie-breaker (C, E4, and E5). All 19 patients with bile duct injury required interventions for management, either singularly or in combination: 10 underwent bile duct reconstruction, six received percutaneous bilioma drainage, and four required biliary stent implantation (Table 3).

**Table 3:** Treatment of patients with bile duct injury (n=19).

Management	n (%)
Bile duct reconstruction	10 (52.6)
Percutaneous bilioma drainage	6 (31.5)
Biliary stent	4 (21)
Right hepatectomy	2 (10.5)
Internal-external biliary diversion	1 (5.2)

## Discussion

Detecting and localizing bile duct lesions is challenging. Clinical manifestations and laboratories are non-specific, and the diagnosis requires high suspicion. In this investigation, it was observed that 15% of patients received an intraoperative diagnosis akin to the findings reported by Vu et al. albeit lower than the estimates reported in the literature, where it is suggested that approximately one-third of patients with Strasberg A-D lesions, and up to 70-80% of patients with Strasberg E lesions, have an intraoperative diagnosis [6,8,17].

Imaging modalities most commonly used to study patients with suspected bile duct injury include ultrasound, CT, MRI, and percutaneous transhepatic cholangiography (PTC) assessment. The first two methods can detect bile duct dilation or perihepatic collections as indirect signs of bile duct injury but do not provide sufficient detail to detect and characterize such lesions. As for PTC, it has a good diagnostic performance, but, like ERCP, it can present complications, including biliary leakage, bleeding, infection, pneumothorax, and even death [8,13,18-20]. Hepatobiliary scintigraphy has good rates of detection of bile duct lesions; however, it does not have the anatomical detail necessary for surgical planning [3,21].

On the other hand, contrasted MRC allows the assessment of biliary anatomy and provides images with functional information that favors the diagnosis of leakage, stenosis, or obstruction. Its performance using T2 sequences is comparable to ERCP for diagnosing multiple bile duct pathologies, such as neoplasms, lithiasis, anomalies, or variants. However, it is limited in the differentiation of collections of biliary origin from other perihepatic collections, considering that both have high signal intensity in the T2 sequences [11,14,22,23]. Then, MRC emerges as the modality of imaging that enables a precise diagnosis of bile duct injuries with a PPV of 100% in studies with hepatospecific contrast and 92% for those with extracellular contrast. Although the small number of the sample does not allow a statistical comparison between the performance of both contrast media, several studies support the theory that the diagnostic effectiveness of MR cholangiography increases with the use of hepatobiliary contrasts [11,13,15,24].

The first published studies on the subject, such as that of Vitellas et al. in 2002, described a good diagnostic performance of MRC contrasted with hepatospecific contrast (sensitivity and specificity exceeding 80%) [25]. Findings congruent to those later published in 2013 by Kantarcı et al. when analyzing conventional T2 and post-contrast T1 potentiated images found a sensitivity of almost 80%, specificity of 100%, and diagnostic yield of 84% [15]. Recent studies, such as Aduna et al. and Cieszanowski et al., have described even better performance with hepatospecific MR cholangiography sensitivity greater than 95% for detecting this type of lesion [13,22].

In agreement with our findings, Alegre et al. included 10 patients with bile duct injury, with a diagnostic accuracy of 100% for MR cholangiography with hepatospecific contrast medium [14]. There was a similar finding to that obtained by Salvolini et al. with 100% performance of contrasted MR cholangiography in detecting bile duct lesions in 22 patients [26]. On the other hand, Kandasamy et al. compared the diagnostic yield of contrasted MR cholangiography in 21 patients, obtaining a PPV of 80% and 94.4% for studies with extracellular and hepatospecific contrast medium, respectively [24].

Although Strasberg type A lesion is the most common post-cholecystectomy form [6,27], in our research, the most frequent types were E2 and E4, with a percentage of 25% each. This distribution aligns with the findings of studies by Wani et al. in 2019, who classified bile duct lesions with MR cholangiography in 25 patients, with types E3 (40%), E2 (36%), and E4 (12%) being the most frequent [28]. In the same year and similarly, Shetty et al. found E2 (50%) and E3 (30.6%) to be the most frequent in 62 patients [29].

ERCP, while valuable diagnostically, is an invasive method with significant drawbacks, including difficulties in accessing the biliary tract, particularly in patients with bilioenteric anastomosis, and complications rates ranging from 1.4% to 3.2% [3], encompassing pancreatitis, sepsis, hemorrhage, duodenal perforation, and death [18]. Notably, one patient in our study received a false-negative diagnosis via ERCP for a Strasberg type E4 lesion, whereas MRI characterized it adequately compared to surgical findings. This disparity usually occurs in cases where complete common bile duct stenosis does not allow visualization of the proximal bile duct, unlike MR cholangiography, which enables the exploration of the bile ducts above and below the level of obstruction, and in those with post-surgical bilioenteric states [21,30]. The disadvantages of contrasted MR cholangiography include limited availability, high cost, sometimes the requisite patient cooperation for apnea, and other contraindications typical of magnetic resonance studies. Moreover, it is a purely diagnostic tool that does not offer a therapeutic alternative [21].

The main strengths of this study are the analysis of diagnostic yield and the use of gold standards for comparison with imaging findings in real clinical conditions. This increases the reliability and validity of the study results. By following the methodology, the study seeks to offer valuable insights into the usefulness of this imaging modality in clinical practice, thus making a substantial contribution to the current knowledge on the subject. The study's limitations predominantly stem from its observational, retrospective, descriptive design and small sample size. Nevertheless, the low incidence of this type of lesion is worth considering, similarly reflected in the small literature series reporting 10 and 28 included patients [5,6,14,25,26,30]. Additionally, it is pertinent to mention the possibility of selection bias when considering a population with a high pretest probability for bile duct injury. This aspect favored the absence of normal (negative) studies. For this reason, it was not possible to calculate sensitivity, specificity, and negative predictive value, which are essential indicators in determining the validity and performance of a diagnostic test.

## Conclusions

Contrasted MR cholangiography is a method with high PPV and almost perfect interobserver agreement for diagnosing bile duct lesions after cholecystectomy. Although the methodological design of this study prevents the obtaining of evidence from which clear guidelines can be generated in the diagnostic approach of the population under study, this is a first step in regional epidemiology and encourages the realization of studies that evaluate the performance of the different methods according to their availability, accessibility, and costs.
